# Physiology of the Inactivation of Vegetative Bacteria by Thermal Treatments: Mode of Action, Influence of Environmental Factors and Inactivation Kinetics

**DOI:** 10.3390/foods6120107

**Published:** 2017-11-30

**Authors:** Guillermo Cebrián, Santiago Condón, Pilar Mañas

**Affiliations:** Tecnología de los Alimentos, Facultad de Veterinaria de Zaragoza, Instituto Agroalimentario de Aragón—IA2, Universidad de Zaragoza-CITA, 50009 Zaragoza, Spain; scondon@unizar.es (S.C.); manas@unizar.es (P.M.)

**Keywords:** heat, inactivation, bacteria, sublethal injury, review

## Abstract

Heat has been used extensively in the food industry as a preservation method, especially due to its ability to inactivate microorganisms present in foods. However, many aspects regarding the mechanisms of bacterial inactivation by heat and the factors affecting this process are still not fully understood. The purpose of this review is to offer a general overview of the most important aspects of the physiology of the inactivation or survival of microorganisms, particularly vegetative bacteria, submitted to heat treatments. This could help improve the design of current heat processes methods in order to apply milder and/or more effective treatments that could fulfill consumer requirements for fresh-like foods while maintaining the advantages of traditional heat treatments.

## 1. Introduction

Heat has been widely used in the food industry as a preservation agent, since it is capable of inactivating most microorganisms and enzymes present in foods. Therefore, heat is a method that can simultaneously guarantee food safety and food stability. 

Heat treatments can be classified into two groups depending on their intensity and their objective: pasteurization and sterilization treatments. Pasteurization treatments aim to inactivate vegetative cells of pathogenic species present in foods; they also extend shelf life, as long as foods are maintained under refrigeration conditions. Sterilization treatments are applied in order to guarantee the stability of the food product at room temperature, an objective that requires the application of temperatures above 100 °C in most cases. Such intense treatments are capable of inactivating microbial spores as well as many enzymes and toxins present in foods, but can also severely modify their organoleptic and nutritional properties.

In this review, we aim to offer a general overview of the most important aspects of the physiology of the inactivation or survival of microorganisms, particularly vegetative bacteria, subjected to heat treatments. Special effort is devoted to the description of the cellular targets affected by inactivation processes, as well as the mechanisms for their repair that bacterial cells are able to trigger. The application of this basic knowledge could help improve the design of current pasteurization processes, leading to milder and/or more effective treatments that could fulfill consumer requirements for fresh-like foods while maintaining the advantages of traditional heat treatments.

## 2. Lethality of Heat on Different Microorganisms

It has been long assumed that the relationship between the number of surviving microorganisms and the time of exposure to a constant temperature is exponential [1]. In order to compare the relative heat resistance of different microorganisms, heat resistance is commonly expressed by the *D_T_* parameter. *D_T_* corresponds to the exposure time, at a given temperature, that is required to reduce the bacterial population by one Log_10_ cycle. In other words, *D_T_* is the time needed to inactivate 90% of the bacterial population. An analogous relationship occurs between the *D_T_* value and treatment temperature. Thus, the *z* value corresponds to the number of degrees required to increase the treatment temperature for reducing the *D_T_* value by one Log_10_ cycle. Although there are numerous exceptions to this behavior, and deviations from the traditional exponential inactivation kinetics are frequently observed (Section 5), *D_T_* and *z* values still provide a useful indicator of the level of heat resistance, and allow for comparisons among different microorganisms and experimental conditions.

Thermal treatments are widely used because of their capacity to inactivate vegetative cells, bacterial spores, yeast and molds. The type of inactivated microorganism depends, of course, on the treatment’s intensity. The degree of heat resistance of different microbial groups varies widely, due to their differing structure and composition, as well as the mechanisms of resistance they are able to develop. The most obvious example of this wide variation is the difference in heat resistance between vegetative cells and spores of the same bacterial species [1]. Thus, bacterial spores are one of the most efficient resistance structures in nature: they are even able to withstand pasteurization treatments (*D_T_* values frequently above 1 min at 100 °C). On the other hand, vegetative yeast cells are generally considered to be more heat sensitive than vegetative bacterial cells [2], although some authors have reported the opposite effect at acid pH [3]. Yeast and molds also show a wide range of heat resistance depending on their structure (sclerotia, hyphae, ascospores, etc.). Ascospores are usually extremely resistant to heat, with *D_T_* values that can reach those of bacterial spores [4].

Table 1 includes the heat resistance parameters of various microorganisms. As pointed out in the introduction, the next sections of this review will focus on vegetative prokaryotic cells, as they are the main target of pasteurization treatments.

The range of heat resistance of vegetative bacterial cells is also wide. Some genera are quite heat sensitive, for instance *Aeromonas* and *Campylobacter* [5,6,7], whereas others are thoroughly heat resistant, such as *Enterococcus* [6,16]. It is generally assumed that Gram-positive cells are more resistant than Gram-negative cells [17], and that coccoid cells are more resistant than bacillary cells [18]. However, these assumptions must be viewed with care, since exceptions have been reported to all these general rules.

Furthermore, differences in heat resistance have been described among different species of the same genus, and among different strains of the same species. Intraspecies variation in resistance has been studied in several microorganisms, including *Escherichia coli*, *Salmonella enterica*, *Aeromonas hydrophila* and *Staphylococcus aureus*, among others [8,19,20,21]. A representative example is *Salmonella enterica* serovar Senftenberg strain 775 W, which shows *D_T_* values 10 times higher than other strains of the same species [22,23]. Many studies have been published on this topic, and some interesting observations have been reported: wild strains, for instance, are frequently more heat resistant than laboratory strains [24].

## 3. Mechanisms of Microbial Inactivation by Heat

### 3.1. General Considerations

The mode of action of heat on microorganisms has been widely studied, and many different cellular alterations have been reported. Before describing the particular alterations that heat can cause in microbial cells, it is important to reflect on the meaning of the terms “living cell” and “inactivated cell”. A practical definition of a living cell is a “cell that has the potential to replicate indefinitely under suitable conditions” [25]. Viability in this context is demonstrated by the cell’s capacity to grow, either in solid media, liquid media, or any other experimental setup. The main objective of food preservation methods is to modify bacterial cells in such a way that they are inactivated: in other words, that they lose the potential to multiply. This modification consists in the alteration of one or more cellular structures or functions, which are generally known as **cellular targets**. In general, the cellular structures or targets that have been most commonly considered as affected by cell inactivation through heat are the outer and inner membrane, the peptidoglycan cell wall, the nucleoid, the cell’s RNA, the ribosomes, and diverse enzymes.

There have been many attempts to identify the particular structures and processes whose alteration, under exposure to heat, leads to cell death. However, given the close and multiple interrelationships between the various structures and cellular functions, interpretation of experimental results is complex. In fact, after decades of research, we must continue to admit that the ultimate cause leading to cell inactivation by heat is not known. Most authors consider that microbial inactivation by heat is a multi-target phenomenon. The relative importance of the damage inflicted to each individual cellular target within the final result—inactivation or survival of the cells of different microbial groups and under differing treatment conditions—is unclear.

This leads to an important concept: the **critical component** [26]. A critical component would be the one whose destruction leads to cell death. It has to fulfill two requirements: it must be indispensable for the self-maintenance and replication of the cell, and it is irreplaceable if rendered non-functional [25]. The first requirement comprises all cellular components that are products of essential genes, whereas the second requirement excludes any component that can be synthesized again after treatment, provided that environmental conditions allow such synthesis. Clear examples of critical components would be RNA polymerase and ribosomes. However, each bacterial cell has an elevated number of ribosomes, and the amount of ribosome loss that a cell can withstand is not clear [27]. Something similar occurs with the cytoplasmic membrane: it is regarded as a critical component, but it has to be damaged to a given degree—enough to prevent its own repair. In addition, the complexity of interactions between different cellular components can be very high. For instance, a destabilization of the ribosomes could occur as a secondary effect of magnesium loss across a slightly damaged membrane [27]. Moreover, these two phenomena—ribosome destabilization and membrane damage—may occur simultaneously, thus preventing a proper interpretation of results.

In summary, the lethality of a thermal treatment will depend on the alteration of at least one critical component beyond a critical threshold, due to the direct effect of heat on the critical target itself, or as the consequence of a parallel alteration of another cellular target (which may not be necessarily lethal).

Moreover, the intrinsic resistance of each particular cell target may vary depending on the type of microorganism and the environmental conditions, i.e., the composition of the treatment medium. Thus, the possibilities (and its level) of alteration of a given cell are diverse. In fact, many intermediate situations occur in practice: for example, cells that present damages in non-critical components and/or in critical components, but at low intensity. This is directly linked to another important phenomenon: cellular **sublethal injury and recovery**. Sublethally injured cells present damages in cellular structures and functions that can only be repaired by the cellular machinery if environmental conditions are appropriate [28]. The practical implications of this phenomenon for food safety are of tremendous importance and are therefore described in detail further below (Section 4.3).

Finally, it is also important to bear in mind that most bacterial species are able to develop **resistance responses** against the stressing action of certain physical and chemical agents. The most typical example is the development of heat resistance after exposure of bacterial cells to sublethal temperatures in the so-called “heat shock response” [29]. Such responses increase the thermostability of various cellular components and/or increase the cellular capacity to repair sublethal injuries [30].

**Figure 1:**
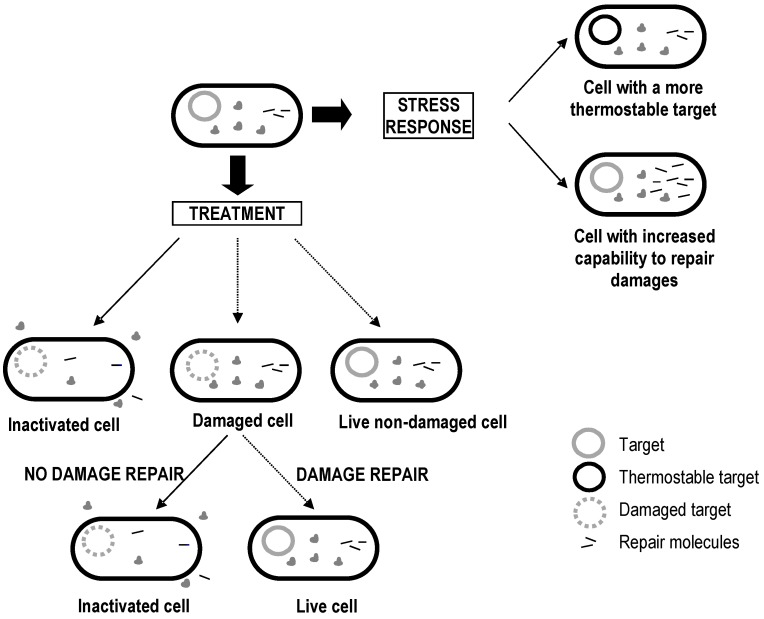
Different scenarios that can determine cellular survival or inactivation upon heat exposure.

Figure 1 presents an outline of the various scenarios that can occur when vegetative bacterial cells are subjected to heat, assuming the simplest situation, i.e., a single target molecule that is also a critical component. 

### 3.2. Effect of Heat on Cellular Targets

The **outer membrane** of Gram-negative cells is one of the structures affected by heat. Damages inflicted to that structure have been evidenced through the sensitization of cells to bile salts, lysozyme or hydrophobic antibiotics [28,31]. The release of lipopolysaccharide molecules and vesicles to the treatment medium has also been reported [32].

The **peptidoglycan wall** is also affected by heat. It has been reported that *S. aureus* cells lose d-alanine from the teichoic acids [28], which leads to the chelation of Mg ions in the wall, preventing their use in certain essential metabolic processes within the cell. Teixeira et al. [33] demonstrated that the cellular wall of *Lactobacillus bulgaricus* was injured after heating at 64 °C, since heated cells were more sensitive to the presence of penicillin.

The role of the **cytoplasmic membrane** in heat inactivation has been studied by several authors. The loss of intracytoplasmic material, such as ions and UV absorbing substances, from heated bacteria has been reported by several researchers in various species [34]. The formation of membrane vesicles and the loss of membrane material have also been frequently described [28,35]. Loss of membrane integrity after heat treatments has also been demonstrated via propidium iodide staining, which is the most widely used marker for membrane integrity [36,37]. Some authors have proposed that the membrane would be related to cell inactivation, since its partial loss of functionality with the consequent loss of internal homeostasis has been demonstrated: alterations in the entrance and exit of several components [36,37,38,39,40], loss of respiration activity [36]; and loss of the osmotic and pH homeostasis [28,33,41,42]. Although the occurrence of membrane alterations in heated cells has been well established, a clear relationship with cell inactivation has not been proven. In fact, various studies indicate that cell death takes place before the membrane becomes permeable to propidium iodide [36,37]. For this reason, it is now assumed that the cytoplasmic membrane is not the only structure involved in cell inactivation by heat. It nevertheless plays an essential role in sublethal damage and recovery phenomena. Furthermore, it cannot be discarded that cellular inactivation could be due to the indirect consequences of subtle damages in the membrane, not detected by propidium iodide staining, on cellular homeostasis, metabolism and also on the integrity of other cellular structures.

The **nucleoid** is an essential molecule for bacterial survival, and its direct implication in the inactivation by other technologies has been demonstrated: for instance, in the cases of ionizing radiation and UV light. In the case of heat treatments, it has been shown that the DNA is one of the cellular constituents with the highest thermostability: its denaturation occurs only at sterilization temperatures, especially under dry heat conditions [1,43]. However, less intense treatments are also capable of damaging DNA in a more subtle way: this has been evidenced through increased mutation frequency in surviving populations after heat exposure [44,45]. Those damages render DNA more exposed to the action of endonucleases, leading to potential subsequent denaturations [45,46,47]. 

DNA alteration might therefore take place during and after heat treatment. This fact is supported by investigations carried out with cells harboring mutations in genes encoding for DNA repair enzymes. Mackey and Seymour [48] showed that *recA*, *recB* and *polA* mutant cells of *E. coli* were more sensitive to heat treatments than their corresponding parental strains. This indicates that heat provokes injuries in the DNA molecule, and that those injuries are repairable. The same authors also reported that the addition of catalase to the recovery medium increased the resistance of the mutant strains to the same level as the parental strains, indicating that the damage to DNA, which was repairable under appropriate conditions, was associated with the presence of reactive oxygen species (ROS). Another important observation is that the final consequences of the damage inflicted to DNA during treatment strongly depend on the degree of enzyme inactivation (catalase, for example).

The effect of heat on **RNA and ribosomes** has also received much attention. These components are more thermosensitive than DNA [49]. RNA denaturation was initially pointed out as one of the most evident consequences of the exposure of bacterial cells to heat [50]. Damage to the ribosomes was also reported [34]. Given the fact that ribosomes consist mainly of RNA and protein, it was predicted that they could be an important critical target, and differential scanning calorimetry (DSC) studies were performed to check this hypothesis [43,51,52,53,54]. Thermograms obtained from whole cells allow researchers to examine the denaturation temperatures of the cellular components, as well as the reversibility of the changes detected. Moreover, a comparison between the thermograms of untreated and heat-treated cells at different intensities permits the identification of the affected components. According to published work, in most cases the irreversible denaturation of the ribosomes occurs at temperatures close to those at which bacteria are inactivated. However, some essential proteins, including the subunits α and β of the RNA polymerase, also denature at similar temperatures [54].

Another aspect of interest in relation with the stability of the ribosomes is the role of magnesium ions, which are known to be essential for the maintenance of the subunits bound to each other. Tolker-Nielsen and Molin [55] demonstrated that the presence of magnesium in the treatment medium protected ribosomes against the action of heat, and increased heat resistance of *S. enterica* var Typhimurium. Thus, it is reasonable to assume that the depletion of magnesium from the cell, for instance as a secondary effect of membrane damage, would cause the destabilization of the ribosomes thereby contributing to cell death.

**Proteins** are distributed among the bacterial cells in almost all cell structures, either as structural proteins or as enzymes. In 1949, Heden and Wyckoff [56] observed through electronic microscopy that the cytoplasm of heat-treated cells presented a granular appearance, which reverted or did not revert depending on the intensity of the treatment. More recently, many authors using different techniques including ultracentrifugation in density gradients and fluorescence spectroscopy/microscopy have demonstrated that heat treatments induce protein denaturation and aggregation in bacterial cells [57,58]. Protein denaturation can lead to a loss of functionality in several ways. Detoxifying enzymes such as catalase or superoxide dismutase, chaperones and proteases (DnaK, DnaJ, GroEl/GroES, Clpx, etc.), as well as DNA repair enzymes, all of which play an important role in the ulterior recovery process, could be relevant targets. Transport pumps and channels are also susceptible to heat denaturation. Moreover, it is reasonable to assume that massive protein aggregation might be difficult for a bacterial cell to counteract if it is already harboring other sublethal injuries, for instance in the membrane. 

Although initial studies discarded the importance of individual proteins as targets for heat inactivation—due to the possibility of resynthesis after treatment, in most cases—they are currently being reconsidered as important potential targets. In particular, it is now being considered that the RNA polymerase could be a realistic critical target [54], although this has not been directly proven.

Apart from the direct effects of heat on cellular components, other indirect phenomena could be involved in cellular inactivation under particular environmental conditions. This is the case of the accumulation of ROS, a phenomenon which has been demonstrated to occur upon heating in *Escherichia coli*, *Cronobacter sakazakii* and *Bacillus cereus* [37,59,60,61]. ROS are able to react with almost all cellular components, including the DNA, lipids from the membrane, and proteins. The origin and actual role of these ROS on cell inactivation is currently not known. Recent studies seem to indicate that, for *E. coli*, formation of ROS would not be the consequence of membrane damage and subsequent electron chain disturbance, since a correlation between envelope permeabilization to propidium iodide and the generation of ROS has not been found [37]. The authors therefore suggested that other phenomena might be the ones responsible for the increase in the amount of ROS: for instance, destabilization of other structures, or loss of activity of bacterial defense systems.

**Figure 2:**
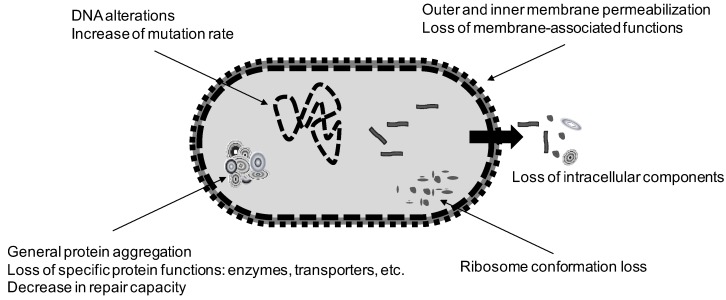
Summary of the most relevant cellular events that occur in a vegetative bacterial cell upon exposure to heat.

Figure 2 summarizes the main events that have been reported to occur in a vegetative bacterial cell exposed to heat. The reader should bear in mind that all these events could somehow be interrelated. One can conclude that heat has the capacity to affect a wide range of structures and cellular functions that lead to cell inactivation under different experimental conditions. This makes heat a multitarget agent that does not exert an “all-or nothing” mode of action. It leads to sublethal damage of cells in different proportions of the population, and their recovery depends on multiple factors such as the intensity of the treatment applied, the treatment medium, the type of microorganism and the conditions for recovery.

## 4. Factors Affecting Bacterial Inactivation

A great number of factors exert an influence on the heat resistance of a given microorganism. In this review we have classified them according to the moment at which they exert their action. The influence of the most important ones is described below.

### 4.1. Factors Acting Prior to Treatment

Growth phase: exponentially growing cells are usually more sensitive to heat than cells in the stationary phase of growth [5,8,16,19,22,62]. This fact is often explained by the increase in the expression of the alternative sigma factors σ^B^ (Gram-positive cells) and σ^S^ (Gram-negative cells) upon entrance into stationary phase. These alternative sigma factors control the transcription of a subset of genes involved in stress resistance, in such a way that some of those genes may play a role in heat survival: for instance, genes involved in DNA stabilization and repair, proteases, and catalases, among others [30,63,64]. 

Growth temperature: it is generally assumed that thermophile microorganisms are more heat-resistant than mesophiles, and these more than psycrophiles, due to their different chemical and structural composition [17]. However, in general terms and for a given microbial species, an increase in growth temperature—within reasonable limits that permit growth or sporulation—also leads to greater heat resistance. This effect has been studied in various species including *Escherichia coli* [9,62], *Listeria monocytogenes* [65], *Yersinia enterocolitica* [5] and *Salmonella enterica* [22]. 

The most generalized explanation of the effect of growth temperature on the heat tolerance of vegetative bacterial cells is that they modify the fatty acid [66] and protein composition [67] of the membrane, thereby adjusting fluidity to maintain the same permeability properties at different temperatures: these changes could be related to heat tolerance. Moreover, cells growing at higher temperatures present a different general protein profile [68,69]; particularly, their content in heat shock proteins (HSPs) is higher. These proteins exert a variety of cellular functions, including well-known assistance in the correct folding and elimination or repair of proteins that may have been damaged by heat, but also the thermostabilization of some structures, such as the membrane [70].

Composition and characteristics of the growth medium: it is generally accepted that an increase in the complexity of the medium leads to an increase in heat resistance [71,72]; however, from the data available, no particular conclusions can be drawn regarding which components are most relevant. It has been reported that a decrease of the water activity of the growth medium protects cells against a subsequent heat treatment [73,74]; a reduction in the pH has a similar effect [75,76,77]. The latter effect seems to depend on the type of acid used [77]. There is no general agreement regarding the mechanism involved in the increase in heat resistance upon exposure to these factors during growth, but a logical explanation would be that they trigger the development of stress adaptation responses in the bacterial cells. 

Exposure to sublethal stresses: bacterial cells are able to develop very effective defense mechanisms against a variety of stressing agents [78]. Many of these defense mechanisms are effective not only against the same agent that triggered them, but also against other agents: this is known as the cross-resistance phenomenon. In the case of heat treatments, the existence of a specific defense response, e.g., the heat shock response, has been long known. It is defined as a homeostatic protective mechanism triggered as a consequence of an increase in the extracellular medium’s temperature. This signal is recognized by cellular sensors, and the transcription of the genes encoding for the HSP (heat shock proteins) is induced. Most of the HSP are proteases and molecular chaperones, whose main function is to eliminate aberrant proteins and to aid in the correct folding of proteins. The direct consequence of their presence is the acquisition of a higher heat resistance, and also resistance to other agents such as ethanol or high hydrostatic pressure, which also induce the misfolding of cellular proteins [79,80,81]. The genetic regulation of the heat shock response has been the subject of numerous investigations in the last decades, and we refer the reader to the excellent reviews that have been published about this topic [69,70,82]. 

Regarding physiological consequences, pioneering studies were carried out with *Escherichia coli* [83] and *Salmonella* spp. [84,85,86]. Later, many other microorganisms, such as *Listeria monocytogenes*, *Lactococcus lactis*, *Lactobacillus plantarum*, *Yersinia enterocolitica* and *Staphylococcus aureus*, were included [87,88,89,90,91,92,93,94]. From published work one can deduce that the increase in heat resistance after application of a heat shock at sublethal temperatures depends on the temperature, time and medium used. A higher temperature (up to a maximum around 45–50 °C) and longer duration (up to 2–3 h) induces greater thermotolerance, and, with regard to the medium, has to contain nutrients that support the development of the response: in other words, very restrictive media prevent the heat shock response [95]. Also, the exposure to increasingly higher temperatures—anisothermic shocks—provokes a thermotolerance acquisition [85,93,94]. The increases in heat resistance reported in literature vary from 2-fold (*D_T_* × 2) to 40-fold approximately, depending on the microorganism and the experimental conditions applied.

There are various physiological reasons for the acquisition of thermotolerance. Firstly, as is explained above, the amount of proteases and chaperones increases notably, thereby preventing and repairing generalized damages to cellular proteins. This, of course, has tremendous implications for cellular survival capacity, since many enzymes and structural proteins are repaired rapidly after treatment. These also include DNA repair enzymes, or the RNA polymerase itself [96]. On the other hand, it has been demonstrated that many HSP are located in the membrane [70] and contribute to its stability and functionality, such as Lo18 in *Oenococcus oenos* [97] or GroEL in *E. coli* [98]. Some of these membrane-bound HSP are involved in the maintenance of enzymes [99,100,101,102], whereas others play a role in modulating the membrane’s mechanical properties [103]. It has also been shown that exposure of bacterial cells to sublethal temperatures causes an increase in the saturation index of the fatty acids of the membrane [104], and that the interaction of some HSP with lipid bilayers increases the membrane’s rigidity [102,105,106], similarly to the effect of an increase in growth temperature.

Also, the ribosomes and RNA seem to be protected from heat shocks. Perhaps the most relevant HSP in this regard is Hfq [107], also known as the “ribosome chaperone”, due to its involvement in preventing and repairing RNA denaturation. The genes encoding DNA repair enzymes also increase their expression after a heat shock [70,108].

Finally, it is important to add that the exposure to other agents prior to heat treatments can also increase the thermotolerance of bacterial cells. This is the case for acid pH [75,94,109], bile salts [109], alkaline pH [110], low water activity [111], and further diverse substances such as β-mercaptoethanol or sodium azide [112]. Particularly relevant in food processing environments, an extreme case of stress adaptation that leads to an increase in heat resistance is that of biofilms [113]. 

From a practical point of view, these resistance responses may represent an increase in the survival capacity of cells exposed to heat treatments: thus, exposure to stresses at sublethal intensity in food processing environments should be avoided whenever possible.

### 4.2. Factors Acting during Treatment

pH of the treatment medium: this factor has been studied in detail, in vegetative cells as well as in spores. Most published investigations conclude that cells are more heat-resistant at pH close to neutrality, although it has been reported that some species, such as *Salmonella enterica* or *Staphylococcus aureus*, are more resistant at pH close to 5.0 [91,114]. Normally, above and below that optimum, heat resistance decreases [18,115]. The type of acid also determines the pH of maximum heat resistance [116]. The reason why cells are more sensitive when heated at acidic pH is not clear. In vegetative cells it is reasonable to assume that those heated in acid media could suffer a rapid cytoplasmic acidification due to the permeabilization of the membrane. This could lead to a more rapid protein denaturation inside the cell. 

From a practical vantage point, the acidification of foods has long been used as a strategy to increase the efficacy of thermal treatments in order to obtain safe and stable foods.

Water activity of the treatment medium: this is another factor of great importance, since the decrease in a_w_ notably increases *D_T_* values in microorganisms. Its influence depends on the species [34], and the protective effect attained is normally of higher magnitude in those organisms which are more sensitive under standard conditions (a_w_ close to 1) [71,117,118]. The type of solute used to decrease water activity is likewise important: in general, solutes such as glycerol, which are permeable to the cytoplasmic membrane, do not impose any osmotic gradient on the cell, and do not provoke the same increase in thermotolerance as non-permeable solutes, such as sucrose [117,118,119]. In any case, the increase in heat resistance is of high magnitude; for example, a decrease in water activity from 0.98 to 0.83 with sucrose causes a 100-fold increase in the *D_T_* value of *Salmonella typhimurium* [119]. 

The mechanism involved in this protection is not fully clear. It is well known that cells suspended in a high-osmolarity medium suffer from a rapid loss of intracellular water (this phenomenon is called plasmolysis), and that the heat stability of proteins increases in media of low a_w_ [72,120]. It has also been suggested that sucrose could interact with membrane phospholipids, increasing the membrane’s heat stability [121].

Treatment medium composition: apart from the intrinsic effect of pH and water activity, the components of the treatment medium also exert an effect on cell survival. It is generally assumed that complex media protect cells against heat inactivation [20,23,122]. The presence of salts in the treatment medium tends to increase bacterial heat resistance. In some cases, this effect could be related to a decrease in a_w_ [123,124], but in other cases it seems to be due to a specific protective effect. This is the case of divalent cations, e.g., Ca^2+^ and Mg^2+^ ions, which are known to stabilize molecules and/or cellular structures including molecular chaperones, ribosomes, the outer membrane, and the DNA molecule [23,55,123]. On the contrary, salts such as phosphates, polyphosphates and nitrites tend to reduce heat resistance [34]. Other compounds such as ethanol [125], or natural antimicrobials such as essential oils or nisin [126,127], also affect heat resistance of bacteria, decreasing it, but their mode of action has not been fully elucidated. Most authors suggest that the antimicrobial substance could somehow interfere with the integrity of the outer and cytoplasmic membrane. 

### 4.3. Factors Acting after Treatment

**Figure 3:**
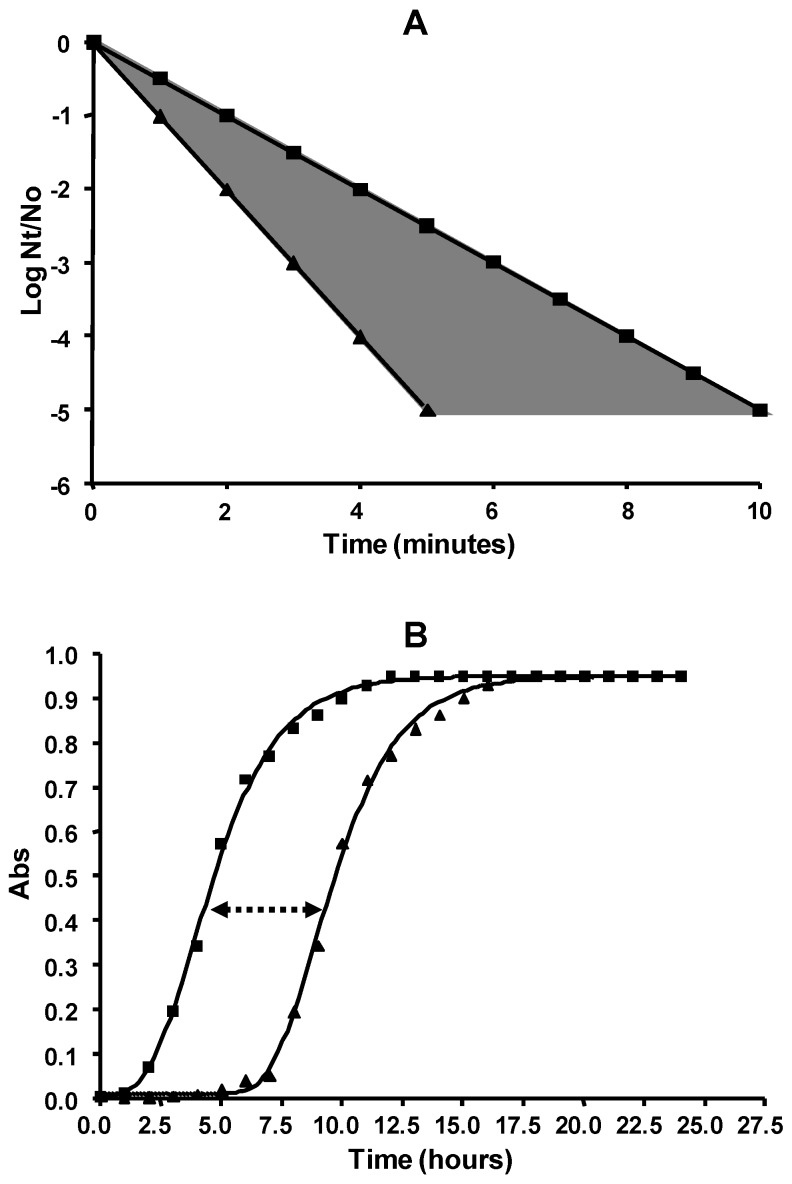
Commonly used experimental approaches to study sublethal heat injury. (**A**) Differential plating technique in non-selective (■) and selective medium (▲): the shadowed area corresponds to the population with sublethal injuries. (**B**) Study of the growth dynamics of non-injured (■) and injured populations (▲) after heat treatment: the lag phase is longer in the latter. N_t_: number of microorganisms at time t. N_0_: number of microorganisms at time 0. Abs: absorbance.

These factors are directly related to the phenomenon of sublethal injury and its repair. The presence of sublethally injured cells—i.e., cells in which certain components have been altered at an intensity that is not sufficient to produce cell death—depends on many factors which include the type of stressing agent applied, the type of microorganism, and the treatment conditions applied. In order to design effective treatments, it is essential to acquire in-depth knowledge of the presence of sublethal damage, its nature, the conditions that induce it, its magnitude, and the conditions which prevent or allow its repair. The presence of sublethally injured cells in a population can be evidenced by several experimental approaches. Perhaps the two most widely used approaches are the study of the differential in colony formation by surviving microorganisms on selective and non-selective media, and the study of the increase of the lag phase during growth in liquid media. The first approach, also called selective plating technique, is based on the principle that cells with sublethal damages should not be able to grow in selective media which contains given inhibitory compounds, such as NaCl or bile salts, whereas they should be able to repair those damages and resume growth in non-selective media, resulting in colony formation in agar plates. Thus, the difference between the number of survivors estimated in the two media will correspond to the sublethally injured cells population. In the second approach, an increase in the duration of the lag phase would be attributed to the time required by bacterial cells to repair damages before returning to normal growth [28]. The fundamentals of both approaches are illustrated in Figure 3A,B, respectively.

The addition of inhibitory compounds to the recovery medium has been used to detect particular cellular alterations in heat-treated cells. For example, it is assumed that the addition of NaCl inhibits the growth of cells with damages in their cytoplasmic membrane, thus unable to control the passage of ions in and out of the cell. Cells with an intact membrane are able to grow in both media, whereas cells with a damaged membrane only grow in the medium without NaCl. Similarly, the addition of acids, alkali and certain ions (Cu, Se) has also been used for the purpose of detecting cells with sublethal injuries in the cytoplasmic membrane, whereas bile salts, lysozyme and some antibiotics have been used to detect damages in the outer membrane or peptidoglycan layer [28]. 

In the case of thermal treatments, given their multitarget mode of action on microorganisms, it is reasonable to expect that sublethal damages of diverse nature and severity will occur within the treated population. In fact, different researchers have reported the occurrence of sublethal damage in practically all cellular components: DNA, RNA, envelopes, enzymes and other proteins. The cells’ repair capacity depends on the presence of repair systems (proteases, chaperones, DNA-repair enzymes, etc.), the activity of which relies on the existence of appropriate conditions in the cytoplasm: pH, osmotic pressure, concentration of essential components, and redox balance. These, in turn, are heavily influenced by environmental conditions.

Temperature during recovery: most authors agree that the optimum temperature for the recovery of damaged cells is lower than that for normal growth [28]. The reason for this effect is not known, although some authors have proposed that lower temperatures would allow a better readjustment of microbial metabolic processes after treatment [128], or that they would allow a more favorable balance between repair processes and degenerative processes leading to cell death [129].

Composition of the recovery medium: as explained above, heat-damaged cells are more sensitive to the presence of potentially toxic compounds in the medium, such as NaCl. For this reason, the heat resistance of a microorganism depends on the composition of the recovery medium. Some components, such as sugars, yeast extract and divalent cations, exert a protective effect [128,130]. However, more complex media do not always favor damage repair [28]. Marcén et al. [37] have demonstrated that recovery of heat-treated *E. coli* cells improved when minimal media instead of nutritionally complex media were used. This effect has been attributed to the presence in complex media of reactive species that emerge as a consequence of autoclaving and exposure to light [28,131].

Apart from the normal components of food that may influence recovery, the presence of antimicrobial substances is of particular practical interest. For instance, the presence of nisin, essential oils or sorbic acid may interfere with recovery processes, adding an extra safety margin to certain thermally-processed foods [132].

pH of the recovery medium: heat-treated cells are able to recover in a narrower pH range than their corresponding non-treated populations [133], and in general, a reduction in the pH of the recovery medium leads to a decrease in the estimated *D_T_* values. 

Atmosphere during recovery: it has been reported that heated *Escherichia coli* O157:H7, *Salmonella enteritidis* and *Listeria monocytogenes* cells recover better under anaerobic or low redox-potential conditions, or in the presence of ROS quenchers [37,65,134], and that the estimated *D_T_* values are higher. This is probably due to a more effective control of ROS production and of ROS-derived damages. Heat treatments may increase the level of intracellular oxidative stress in many ways: for instance, via the thermal inactivation of ROS-detoxifying enzymes (catalases, superoxide dismutase, alkylperoxidases), via the loss of important reduced thiols through a permeabilized membrane (gluthatione), or via perturbations in the normal function of the electron transport chain with the subsequent increase of internally-produced ROS (among other possibilities).

Despite the fact that much effort has been directed in recent years to acquire more thorough knowledge of the occurrence of sublethal injury and its repair, it is still an important physiological factor which, in our opinion, remains underestimated and deserves more attention.

## 5. Biological Basis for the Kinetics of Inactivation by Heat

**Figure 4:**
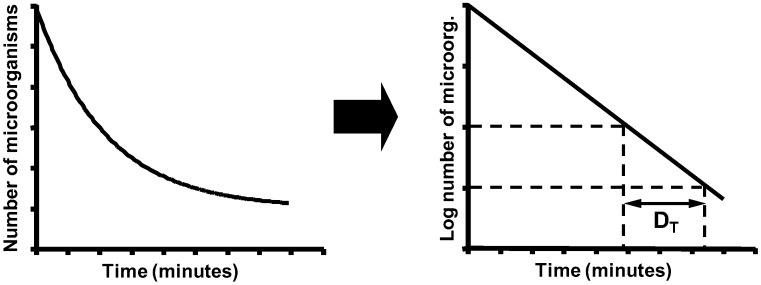
Effect of treatment time on microbial survival to a heat treatment at constant temperature.

Bigelow and Esty [135] were the first researchers who carried out a systematic study to describe how microorganisms are inactivated when they are exposed to heat. Their experiments led to the conclusion that, at constant temperature, after equal time intervals, the percentage of cells in the bacterial population that is inactivated is constant. In other words, the number of microorganisms surviving exposure to thermal treatment is an exponential function of exposure time (Figure 4). This allows us to calculate the *D_T_* value parameter as the inverse negative of the slope of the survival curves, which represent the Log_10_ of the number of survivors versus treatment time.

According to Gould [1], the theory most widely accepted as an explanation of the exponential inactivation kinetics is based on the principles of thermodynamics, analogous to the Arrhenius equation. This theory assumes that all the microbial cells in the population are equally sensitive to heat, and that the water molecules surrounding the cellular targets have a Boltzmann-like distribution of energies. Under these conditions, inactivation kinetics would follow an exponential curve if cellular inactivation were due to a lethal hit on a single target, which should be present as only one or very few copies inside the cells, and if the inactivation were an all-or-nothing effect with no possibility of target repair. In other words, and according to this theory, the inactivation kinetics is the reflection of a probabilistic phenomenon: as a target structure involved in cell inactivation, DNA would be the most probable candidate [1].

**Figure 5:**
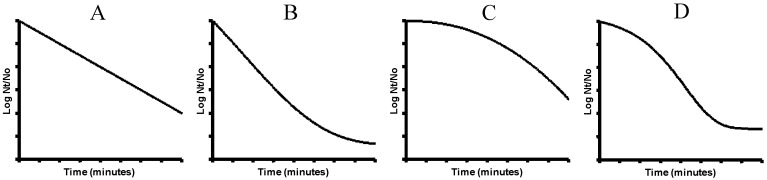
Profiles of heat survival curves. (**A**) Linear profile; (**B**) presence of tail, concave profile; (**C**) presence of shoulder, convex profile; (**D**) tail and shoulder, sigmoid profile.

However, evidence has emerged that does not support this theory. On the one hand, it has been demonstrated that many different cellular structures are damaged after exposure to heat, and that those damages could individually lead to cell death. On the other hand, it has been shown that DNA is only extensively damaged at high temperatures, well above the temperatures that are lethal for vegetative cells. Moreover, we know that heat inactivation is by no means an all-or-nothing effect: sublethal injury is almost invariably detected. Perhaps these inconsistencies may explain that, although in some occasions linear survival curves are observed, the presence of deviations, i.e., shoulder and tail phenomena, is quite frequent (Figure 5).

The **shoulder** phenomenon could be due to simple methodological artifacts (cell clumping, heterogeneity of the treatment, etc.), but physiological reasons could also explain its occurrence. In most cases, shoulders are attributed to the presence of cells with sublethal injuries of increasing severity during the first instants of treatment. Until the amount and severity of the damages surpass a given threshold, the microbial count does not start to drop. 

The **tailing** phenomenon is even more important from a practical point of view, since the possible presence of a small percentage of cells capable of surviving a thermal treatment represents a risk to food safety and stability. In many cases, tails are attributed to methodological causes such as a heterogeneity of heat distribution; however, factors related to cell physiology may likewise explain the presence of tails. These basically include the existence of a distribution of intrinsic thermotolerance within the bacterial population, the presence of microbial adaptation phenomena occurring during treatment, even at lethal temperatures [86], and the existence of multitarget inactivation phenomenon.

Such deviations occur frequently. This, together with the difficulties involved in attempting to propose an adequate biological interpretation of the classical kinetics, and the fact that it is virtually impossible to have a microbial population whose constituent individuals are exactly equal, i.e., with the same thermotolerance and under exactly identical physical–chemical conditions, has led many researchers to question the validity of the traditional model, and to propose alternatives [136]. 

The different primary models applied to heat microbial inactivation, i.e., equations that describe the variation of the number of microorganisms versus heating time, proposed so far as an alternative to the traditional exponential kinetics can be classified into mechanistic or empirical models depending on the existence of a biological basis behind the mathematics. Empirical models are mathematical equations developed for any application whatsoever, which accurately describe the data obtained under particular experimental conditions. This would be the case, for instance, of the modified Gompertz equation [137], which describes a sigmoidal curve with an upper and a lower asymptote. Conversely, mechanistic models are created with the purpose of reflecting physiological aspects of microorganisms, in order to have a better predicting capacity. They are generally more complex; however, most researchers in the field agree upon the convenience of developing such models, which reflect the physiology of inactivation by heat at the cellular level [20,138]. Most of the models currently available are in fact a mixture of both: empirical and mechanistic. It is not the purpose of this review to explain the different models or equations in detail: the reader is referred to the publications of Geeraerd et al. [139], Cappuyns et al. [140] and Smelt and Brul [141], which analyze many relevant models in detail (the classical thermal death model, biphasic models, the modified Baranyi model, the Sapru model, the critical sites model, etc.).

Examples of models that have recently been gaining popularity among researchers are the Weibull-like models [142,143], as well as the model developed by Geeraerd et al. [139]. The Weibull-like models are equations that accurately describe straight, concave upward and convex curves. These Weibull-like equations describe the survival curve as a reflection of the distribution of individual resistance among the microbial cells within a population. The Geeraerd model also provides good fits to a variety of curves [138], and the parameters that define the equation may be more directly interpreted as holding biological significance: *Sl* (shoulder length, min); *K_max_* (maximum inactivation rate, min^−1^, which is a parameter based on first-order kinetics in the linear portion of the survival curve). The level of the tail (Log cfu/mL), which is calculated based on the assumption of the existence of a heat-resistant subpopulation, can also be estimated if needed. These kinds of models (Geeraerd and Modified Baranyi, among others) permit researchers to describe and quantitatively compare inactivation kinetics, and attribute the differences to particular physiological facts, such as, for instance, the presence of recovery mechanisms that give rise to a shoulder.

**Figure 6:**
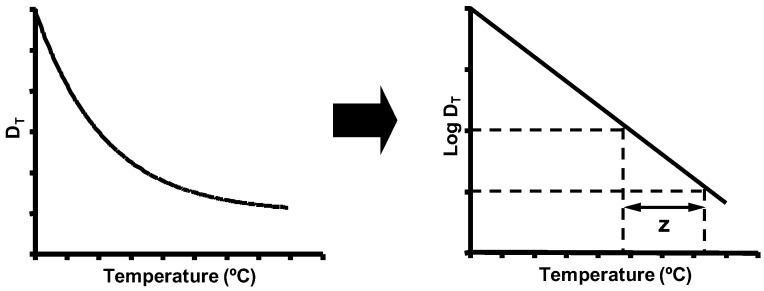
Influence of treatment temperature on the lethality of heat (*D_T_* values).

Secondary models are equations that describe how the parameters obtained from the primary model tend to change with environmental conditions: treatment temperature, water activity, pH of the treatment medium, etc. The most typical example is the secondary model that describes the exponential relationship between the *D_T_* value and treatment temperature, already developed by Bigelow and Esty (Figure 6). This approach is also frequently applied to the parameters obtained with primary models which do not describe exponential kinetics. Thus, it is a useful tool for comparing the resistances of different microorganisms and the effects of different heating media with one another.

Mathematical models are necessary for the calculation and adjustment of thermal treatments applied by the industry in order to guarantee the desired level of inactivation of the target microorganism with minimum side effects on the sensory and nutritional attributes of food. However, it is difficult to develop models that are sufficiently comprehensive. No single model currently includes all the intrinsic and extrinsic variables that may affect microbial inactivation by heat. Finally, it is also remarkable that, as pointed out repeatedly in this article, many aspects still require further investigation. In recent years, much research in food preservation methods has focused on novel, non-thermal technologies. Although the field of heat inactivation has profited from some of the developments in this area—particularly from the development of software tools and mathematical models to describe the kinetics of inactivation—many physiological aspects still deserve further attention, given the complexity of microbial inactivation by heat.

## 6. Conclusions

Thermal treatments are extensively used in the industry to pasteurize and sterilize different foods. Microbial resistance to thermal treatments varies widely among species, and even within the same species. The reasons behind this behavior are not always known, and admittedly the final events leading to bacterial cell inactivation by heat are not fully clear. Heat affects diverse cellular structures and functions to a different degree, and those structures are interlinked. This complexity leads to the presence of sublethally damaged cells with a variety of injuries, which will only be able to recover and resume growth under appropriate environmental conditions. In addition, the survival or inactivation of vegetative bacteria exposed to thermal treatments is influenced by many factors, such as growth conditions (temperature, time, composition of the growth medium), previous exposure to stresses, physicochemical characteristics of the treatment medium, and environmental conditions after the treatment. The influence of these factors may be of great magnitude.

A more in-depth knowledge of the microbial physiological aspects related to heat inactivation and/or survival is needed to avoid process conditions that favor cell survival and recovery, and conversely, to select conditions that increase treatment lethality. Previous exposure to conditions that can trigger the development of resistance responses should be avoided. In addition, the efficacy of heat treatments might be increased by an adequate design of combined processes, taking advantage of the occurrence of sublethal injury. This phenomenon can increase the lethality of some inhibitory compounds, for instance through an increase of envelopes permeability to antimicrobials such as nisin or lysozyme. Besides, the design of combined processes can also be based on the ability of some compounds to inhibit the mechanisms of heat damage repair, thus leading to the inactivation of sublethally injured cells. Moreover, the development of mathematical models that permit the calculation of processing parameters that take cell physiological factors into account is also needed. This knowledge would help improve current thermal treatments in order to obtain safer foods by applying milder thermal treatments.

## Figures and Tables

**Table 1:** Range of *D_T_* values of different bacterial species in buffers and foods (pH range 5.5–7.0; a_w_ > 0.98). Sources [5,6,7,8,9,10,11,12,13,14,15].

	Bacterial Species	Temperature (°C)	*D_T_* (Minutes)	*z* (°C)
**Vegetative cells**	*Aeromonas hydrophila*	60	<0.02	5.2–7.7
	*Campylobacter* spp.	60	<0.01–0.11	4.1–4.7
	*Yersinia enterocolitica*	60	0.07–0.8	4.0–5.8
	*Salmonella enterica*	60	0.1–3.3	3.8–6.3
	*Cronobacter sakazakii*	60	0.05–2.0	4.1–6.2
	*Escherichia coli*	60	0.7–2.7	3.2–5.2
	*Staphylococcus aureus*	60	0.2–6.0	3.6–8.5
	*Listeria monocytogenes*	60	0.5–15	5.2–5.8
	*Enterococcus faecium*	60	5.0–30	4.3–8.0
**Spores**	*Bacillus subtilis*	100	3.31–>100	6.7–10.1
	*Clostridium botulinum* (*proteolytic*)	121	<0.01–0.22	7.6–12.1
	*Geobacillus stearothermophilus*	121	0.1–5.0	7.3–12.2
